# Screening and predictive analysis of common regulatory genes in diabetes and rectal cancer: transcriptional and drug modulation mechanisms

**DOI:** 10.1186/s40001-025-03640-x

**Published:** 2025-12-10

**Authors:** Shuxing Wu, Hongjie Huo, Yuansheng Liu, Xinyang Shi, Chunhui Liu, Hanyi Zha, Yang Shi

**Affiliations:** 1 Department of Cardiology 1, Tianjin Union Medical Center, The First Affiliated Hospital of Nankai University, Tianjin, 300121 China; 2 Department of Respiratory Medicine, Tianjin Union Medical Center, The First Affiliated Hospital of Nankai University, Tianjin, 300121 China; 3 Department of Colorectal Surgery, Tianjin Union Medical Center, The First Affiliated Hospital of Nankai University, Tianjin, 300121 China; 4 Tianjin Institute of Coloproctology, The First Affiliated Hospital of Nankai University, Tianjin, 300121 China; 5 School of Medicine, Nankai University, 94 Weijin Road, Tianjin, 300071 China; 6 North China University of Science and Technology Affiliated Hospital, Tangshan, 063000 China

**Keywords:** Type 2 diabetes mellitus, Rectal cancer, Hub gene, Bioinformatics, Drug molecule, Disease association

## Abstract

**Background:**

There is a strong association between type 2 diabetes mellitus (T2DM) and rectal cancer (RC), but a detailed understanding of the potential mechanistic links and gene regulatory interactions between these two diseases remains limited.

**Methods:**

The differential expression analysis and RT-qPCR were performed on the expression data of T2DM and RC to identify shared differentially expressed genes (DEGs) between these diseases. Functional enrichment and protein–protein interaction (PPI) analyses were conducted. Subsequently, the Cytoscape software was used to calculate the protein centrality PPI network diagram and screen hub genes. Predictive analyses were performed for upstream transcription factors (TFs) of these hub genes to uncover potential regulatory mechanisms. Additionally, candidate TFs were analyzed for modulation of upstream drugs, including molecular docking studies.

**Results:**

The expression data analysis of T2DM and RC identified 17 significantly upregulated and 111 significantly downregulated genes in both diseases. Further PPI analysis and centrality calculation highlighted 15 genes, including SYP, as central within the interaction network, suggesting their key regulatory roles in T2DM and RC. In addition, we used RT-PCR to detect the expression of several DEG genes in T2DM and RC, and the results showed that CDH3, CHGA, ESR1, SCG3, SP1 and SYP genes were the highest in patients with comorbidities, while compared with normal healthy people, the expression of CDH3, CHGA, ESR1, SCG3, SP1 and SYP genes was higher. The expressions of CDH3, CHGA, ESR1, SCG3, SP1 and SYP genes were also significantly increased in T2DM and RC, which was consistent with our prediction. Transcription factor prediction indicated that SP1 could regulate multiple hub genes, suggesting its pivotal regulatory role in T2DM and RC. Further molecular docking revealed that SP1 may target genes for stearic acid and (−)-epigallocatechin-3-gallate (EGCG).

**Conclusions:**

Genes such as SYP show significant differential expression in both T2DM and RC and may play significant roles in the progression of T2DM and RC. Transcription factor analysis suggested that SP1 could regulate multiple hub genes. Molecular docking indicated that SP1 is a potential target for active components like stearic acid and EGCG in *Ginkgo biloba*, highlighting its potential as a therapeutic agent for T2DM and RC.

**Supplementary Information:**

The online version contains supplementary material available at 10.1186/s40001-025-03640-x.

## Introduction

Diabetes and cancer are among the leading causes of death worldwide. Global health statistics from 1990 to 2013 indicate a 90% increase in the mortality rate from diabetes, with colorectal cancer (CRC) deaths rising by 57% during the same period [1]. Studies have found that diabetes is usually accompanied by the occurrence of diseases such as hypertension and kidney disease, and has obvious adverse effects on patients [2, 3]. Recent studies and meta-analyses confirm that diabetes increases the risk of several solid and hematological malignancies, such as liver, pancreatic, CRC, and lymphomas [4]. Historical data from 1984 also show a marked increase in diabetes prevalence among patients with CRC [5], supporting the hyperinsulinemia hypothesis, which posits that high serum insulin levels may promote colon tumor growth and act as a mitogen, thereby increasing CRC risk [6]. Research conducted by Yang et al. has revealed that insulin therapy may further increase the risk of CRC in patients with type 2 diabetes mellitus (T2DM) [7]. Additionally, recent studies have identified common risk factors for both diabetes and CRC, potentially increasing the risk of CRC in diabetic patients [8–14].

T2DM is the most prevalent form of diabetes, and although numerous studies have suggested a close link between the incidence of diabetes and RC, the direct regulatory mechanisms underlying this association remain unclear. Research by Liu et al. has identified that miR-98 can target IGF1R and inhibit tumor proliferation and invasion in T2DM compounded with CRC [15]. Additionally, a study by Wu et al. has indicated that platelet-endothelial cell adhesion molecule 1 (PECAM-1) may influence the carcinogenic progression and development of CRC in diabetic patients [16]. While current research supports a strong association between T2DM and CRC, a detailed understanding of the potential mechanistic links and gene regulatory interactions between these two diseases is still limited. By analyzing gene expression data from T2DM and RC, our study aims to identify key regulatory genes shared between these conditions and predict their potential regulatory mechanisms. It provides new research directions and a theoretical basis for a deeper understanding of the regulatory relationships between T2D and RC. Furthermore, this study predicts upstream drug molecules for hub genes, offering new insights into potential therapeutics for the combined disease phenotype of T2DM and RC.

## Materials and methods

### Data preparation and analysis of differential gene expression

Using the Gene Expression Omnibus (GEO) database (https://www.ncbi.nlm.nih.gov/geo/), we downloaded the microarray GSE76894 of T2DM, including data from 84 normal and 19 T2DM samples. We downloaded gene expression data from the Cancer Genome Atlas (TCGA) GDC database (https://portal.gdc.cancer.gov/) for ten normal and 167 RC samples. Differential gene expression analysis was conducted using the "limma" package in R, with normal samples as controls. The *p*-values were adjusted using the false discovery rate (FDR) method, selecting differentially expressed genes (DEGs) with an absolute fold change (|FC|) greater than 1.5 and an adjusted *p*-value (adj.*p*.val) below 0.05.

### Functional enrichment of DEGs

The "clusterProfiler" package in R was utilized to perform Gene Ontology (GO) and Kyoto Encyclopedia of Genes and Genomes (KEGG) pathway enrichment analyses on genes exhibiting significant differential expression in both T2DM and RC [17–20]. The GO enrichment included cellular components (CC), biological processes (BP), and molecular functions (MF), analyzed using the enrichGO function with a significance threshold of FDR < 0.05. KEGG pathway enrichment was conducted using the enrichKEGG function. The "Circlize" package was employed to create circular diagrams of GO and KEGG pathway enrichments.

### Protein–protein interaction (PPI)

PPI analysis was performed on genes with similar expression trends in T2DM and RC using the STRING database (https://cn.string-db.org/). The interaction network data were visualized using Cytoscape v3.9.1. Clustering within the PPI network was conducted using the MCODE plugin [21]. During the clustering process, the degree cutoff was set to 2, the Node Score Cutoff was set to 0.2, and the K-core was set to 2. Subsequently, the centrality of each gene was calculated using the CytoHubba plugin [22]. Previous studies utilizing this plugin have suggested that the Maximal Clique Centrality (MCC) algorithm is superior to other methods, such as the density of maximum neighborhood component (DMNC) in identifying hub genes [23]. Therefore, MCC was selected as the method for calculating the hub scores. The MCC score of each gene in the protein network graph was calculated after computation, and the top 15 genes with the highest MCC scores were selected for subsequent analysis.

### GeneMANIA prediction

The GeneMANIA database was used to predict related genes and their functions for the candidate genes identified [19]. In the prediction process, the maximum number of genes was selected as 50, the minimum number of genes was selected as 10, and query-dependent weighting was selected as the automatically selected weighting method. All networks were selected during the prediction, and the functions displayed were related to DNA and cell replication.

### Prediction of transcription factors

Predictions for upstream transcription factors (TFs) of the candidate genes were performed using the TRRUST V2.0 database (https://www.grnpedia.org/trrust/) [24]. Meanwhile, the transcription binding domains of core transcription factors on candidate genes were predicted through the JASPAR database (https://jaspar.elixir.no/). The top 15 genes with the highest MCC scores in the PPI network were selected for analysis to identify additional relevant transcription factors. Results were downloaded and visualized as transcription factor–target gene regulatory networks using Cytoscape v3.9.1.

### Drug prediction and molecular docking

Potential drugs and active components targeting the hub TFs were identified using the TCSMP database (https://old.tcmsp-e.com/tcmsp.php). The search type was set to "Target name", while other parameters were kept at their default settings during the database search. Small molecule ligands’ 2D structures were retrieved from the PubChem database (https://pubchem.ncbi.nlm.nih.gov/), and protein receptor molecular structures were obtained from the PDB database (https://www.rcsb.org/). Protein structures were processed using PyMol software to remove water molecules and small molecule ligands. Molecular docking was performed using Vina software, predicting interactions between active drug molecules and protein receptors. Results were visualized using PyMol software, generating diagrams of the molecular docking structure.

### Real time polymerase chain reaction (qRT-PCR)

RNA extraction and reverse transcription: Total RNA was extracted from tissues using TRIzol reagent (Invitrogen, USA) according to the manufacturer’s instructions. All the clinical tissues we obtained were from the Oncology Department of The First Affiliated Hospital of Nankai University. All the patients (*n* = 6, equal numbers of males and females, patients with T2DM, RC, comorbidities of T2DM and RC and healthy population) and their families were informed. Moreover, all of our clinical trials passed the clinical trial ethics (NO. 2024-B43). RNA concentration and purity (A260/A280 ratio of 1.8–2.0) were measured using a NanoDrop spectrophotometer (Thermo Fisher Scientific, USA). For reverse transcription, 1 µg of total RNA was reverse-transcribed into cDNA using a PrimeScript RT Reagent Kit (Takara, Japan) under the following conditions: 37 °C for 15 min and 85 °C for 5 s.

RT-PCR procedure: Real-time PCR was performed using SYBR Green Master Mix (Takara, Japan) on a QuantStudio 5 Real-Time PCR System (Applied Biosystems, USA). The PCR protocol included an initial denaturation step at 95 °C for 5 min, followed by 40 cycles of 95 °C for 10 s and 60 °C for 30 s. The GAPDH was used as the internal control. The relative mRNA expression levels of target genes were calculated using the 2^−ΔΔCt^ method. More detail about qRT-PCR analysis was performed as previously study described. The experiments and methods were performed in accordance with relevant guidelines and regulations of The First Affiliated Hospital of Nankai University. The informed consent was obtained from all subjects and their legal guardians.

The primer sequences of DEG are as follows(Table 1).

**Table 1:** Primer sequences used in the RT-PCR experiment (*n* = 6)

Primer		Sequence (5′−3′)	Size (bp)
*CDH3*	F	CAGCGACGGAAAGAGTATGAGC	204
	R	AATGTGGGCAACCTGGGAGTAG	
*CHGA*	F	AGGAGCCAGGGAGTAACGAAGAG	208
	R	TGAGGAACTGTGGAGAGACGGTG	
*SP1*	F	CCTGAAGAAAAGATTCCAGACGATG	143
	R	TCAAAGTAGGTGGTGAAGGGCTC	
*SYP*	F	TGCCAAATACTTTCTCCATCAATCTC	127
	R	CCAGTCATCACGGTCAGGTTTC	
*ESR1*	F	GGGACTAACTCCTCCGACCTTC	229
	R	GCTGGTAGAGTCGGAGAAGTTGAG	
*SCG3*	F	TGAACGGTGGTGTGTTTGCTGTC	111
	R	CTGGTAGAGGAGTGTGCTTGCGG	
*β-actin*	F	TGGCACCCAGCACAATGAA	167
	R	GAAGCATTTGCGGTGGACG	

### Statistical analysis

Experimental data were analyzed using SPSS 25.0 software. Measurement data were expressed as mean ± standard deviation (mean ± SD). Comparisons between groups were performed using Student’s t-test or one-way analysis of variance (one-way ANOVA). All sample sizes/experiments were repeated at least 3 times. The *p*-value < 0.05 was considered statistically significant. The correction for multiple testing in experimental validation was utilized.

## Results

### Differential genes expression analysis

**Fig. 1:**
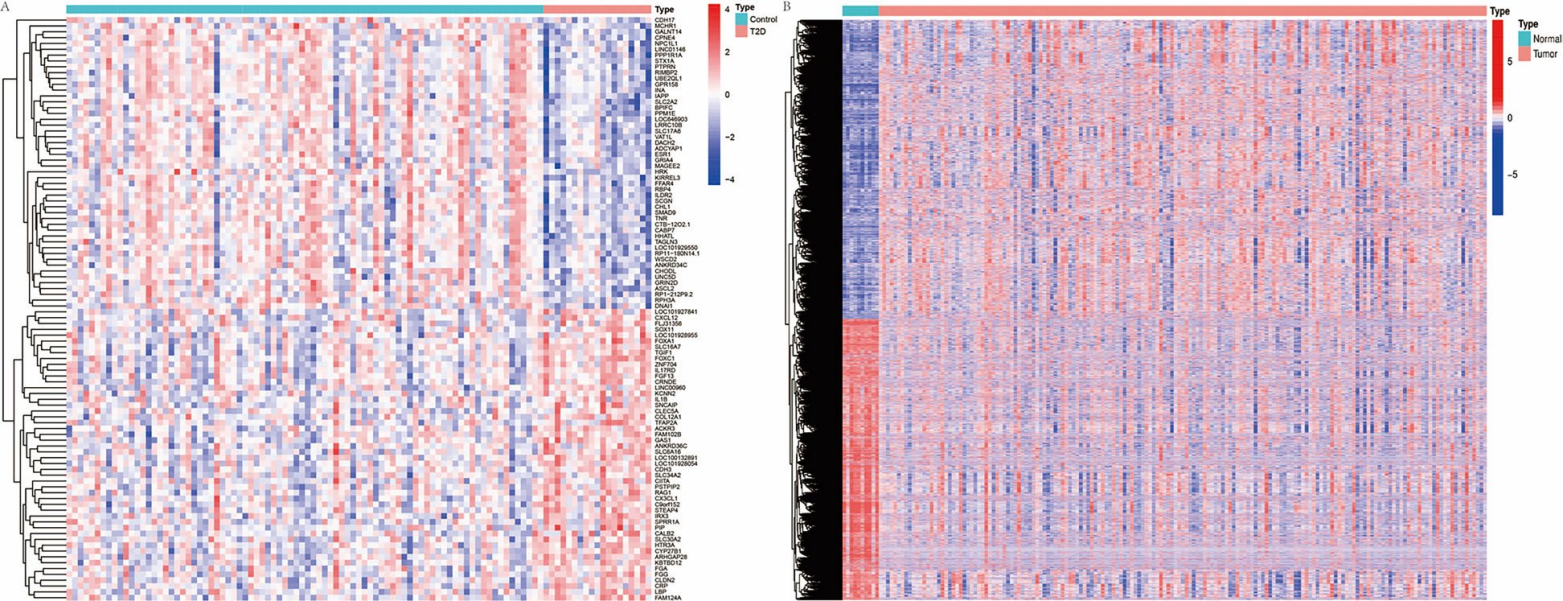
Heatmaps of DEGs in diabetes and colorectal cancer. **A** Heatmap of DEGs from the diabetes microarray GSE76894; **B** heatmap showing DEGs in colorectal cancer and normal samples from the TCGA database

Differential gene expression analysis of the T2DM microarray GSE76894 yielded 273 significant DEGs (Fig. 1A, Supplementary Table 1), with 71 genes significantly upregulated and 202 genes significantly downregulated. In addition, differential gene expression analysis of RC samples from the TCGA database revealed 13,828 DEGs (Fig. 1B, Supplementary Table 2), with 7125 genes upregulated and 6703 genes downregulated in RC.

### Identification and verification of shared DEGs

**Fig. 2:**
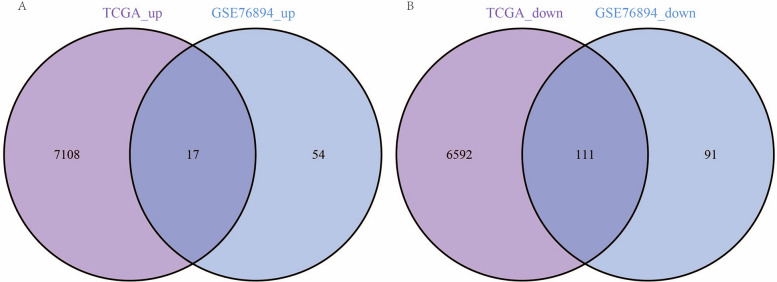
Shared DEGs in diabetes and colorectal cancer. **A** Venn diagram displaying genes that are significantly upregulated in both colorectal cancer and diabetes, with the central portion representing genes common to both datasets; **B** Venn diagram showing genes that are significantly downregulated in both diseases, with the central portion indicating genes shared across both diseases

We intersected significantly upregulated genes from both diseases to identify candidate genes that may play regulatory roles in T2DM and RC. This analysis found 17 genes consistently upregulated across both diseases (Fig. 2A, C). In addition, the intersection of significantly downregulated genes in both diseases revealed 111 genes consistently downregulated (Fig. 2B, C). This finding suggests that these 128 genes may play important regulatory roles in both diseases.

### Functional enrichment of DEGs

**Fig. 3:**
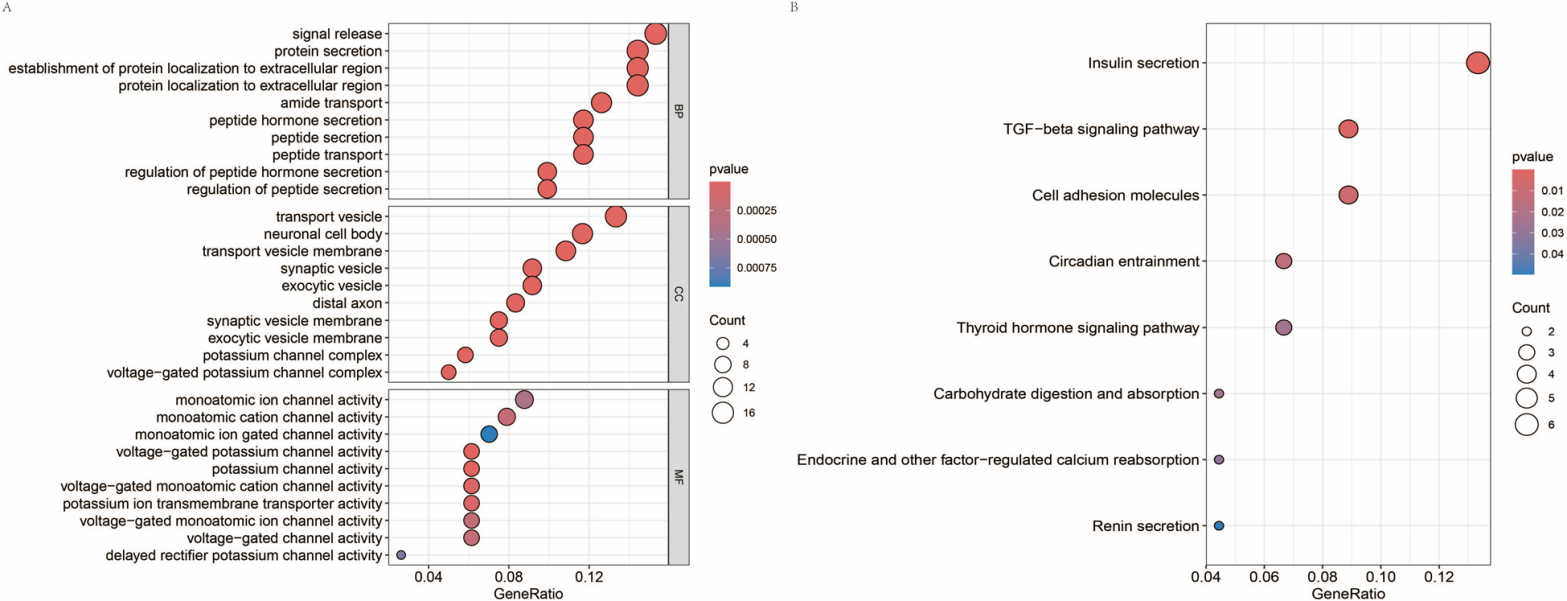
Functional enrichment analysis of shared genes. **A** GO enrichment, where the x-axis represents the GeneRatio, the y-axis represents GO terms, and the histogram on the right displays a color gradient; **B** pathway enrichment

Further functional enrichment analysis was conducted on the 182 genes showing significant shared DEGs in both diseases. GO enrichment analysis indicated that these genes were predominantly enriched in functions related to "signal release", "transport vesicle", and "monoatomic ion channel activity" (Fig. 3A, Supplementary Table 3). KEGG pathway analysis revealed significant enrichment in pathways such as "Insulin secretion" and "TGF-beta signaling pathway" (Fig. 3B, Supplementary Table 4). Given the crucial role of insulin metabolism in T2DM and the TGF-beta signaling pathway in various cancers, these findings suggest that these DEGs are important for the progression of both diseases.

### PPI

**Fig. 4:**
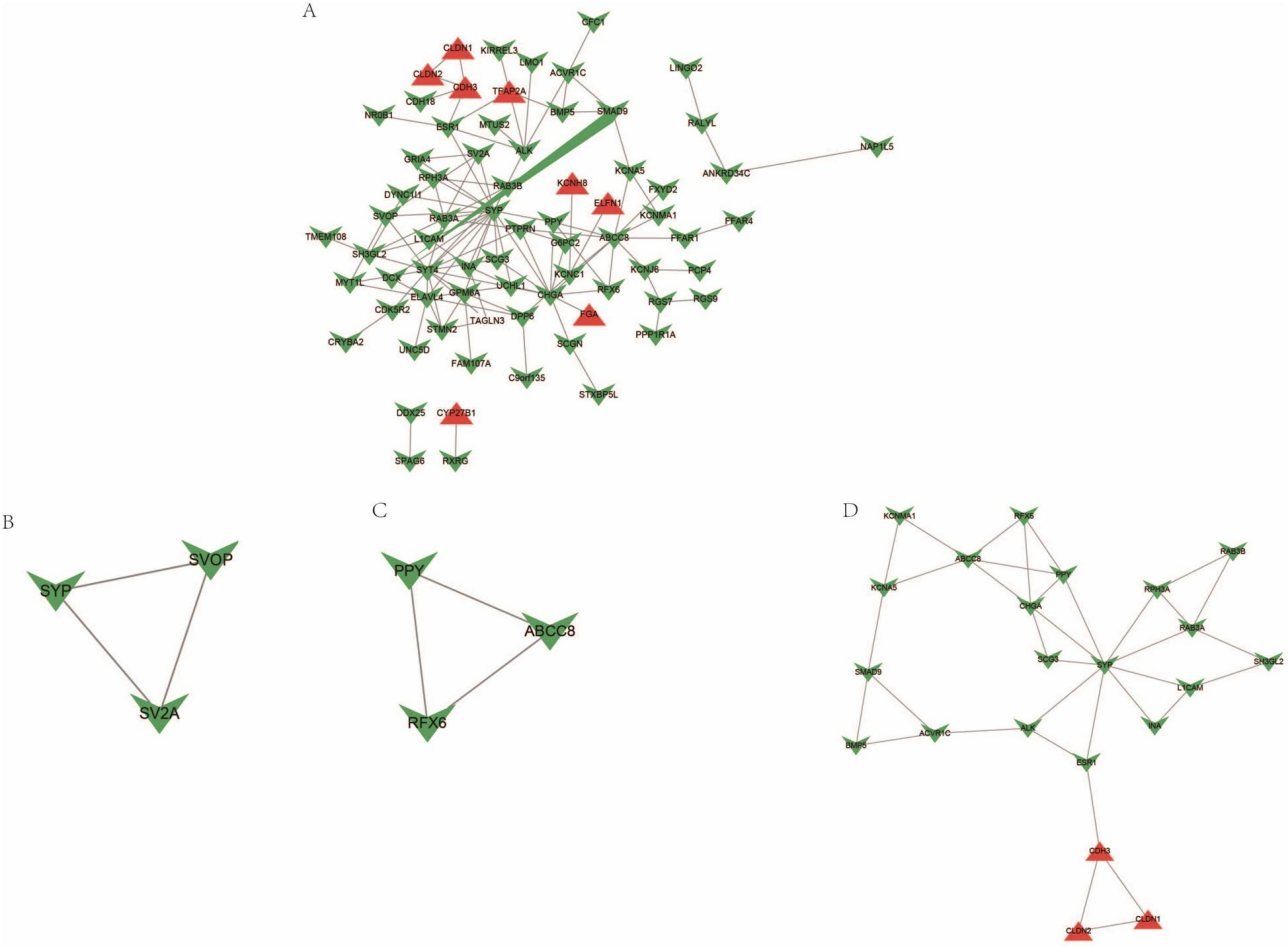
PPI network of shared genes. **A** PPI analysis of shared DEGs, where red indicates genes upregulated in both diseases and green denotes genes downregulated in both. Lines between proteins indicate interaction relationships. **B**, **C**, and **D** Clusters within the PPI network were identified using the MCODE plugin in Cytoscape, where (**B**, **C**), and (**D**) represent two distinct subnetwork clusters

PPI analysis of the 182 candidate DEGs revealed extensive interactions, predominantly among genes consistently downregulated in both diseases (Fig. 4A). Clustering within the PPI network identified three distinct groups (Fig. 4A–D). Notably, most interacting genes were those significantly downregulated in both T2DM and RC, and most clustered genes were significantly downregulated in the PPI clustering, indicating a potentially critical regulatory role in these diseases.

### GeneMANIA prediction

**Fig. 5:**
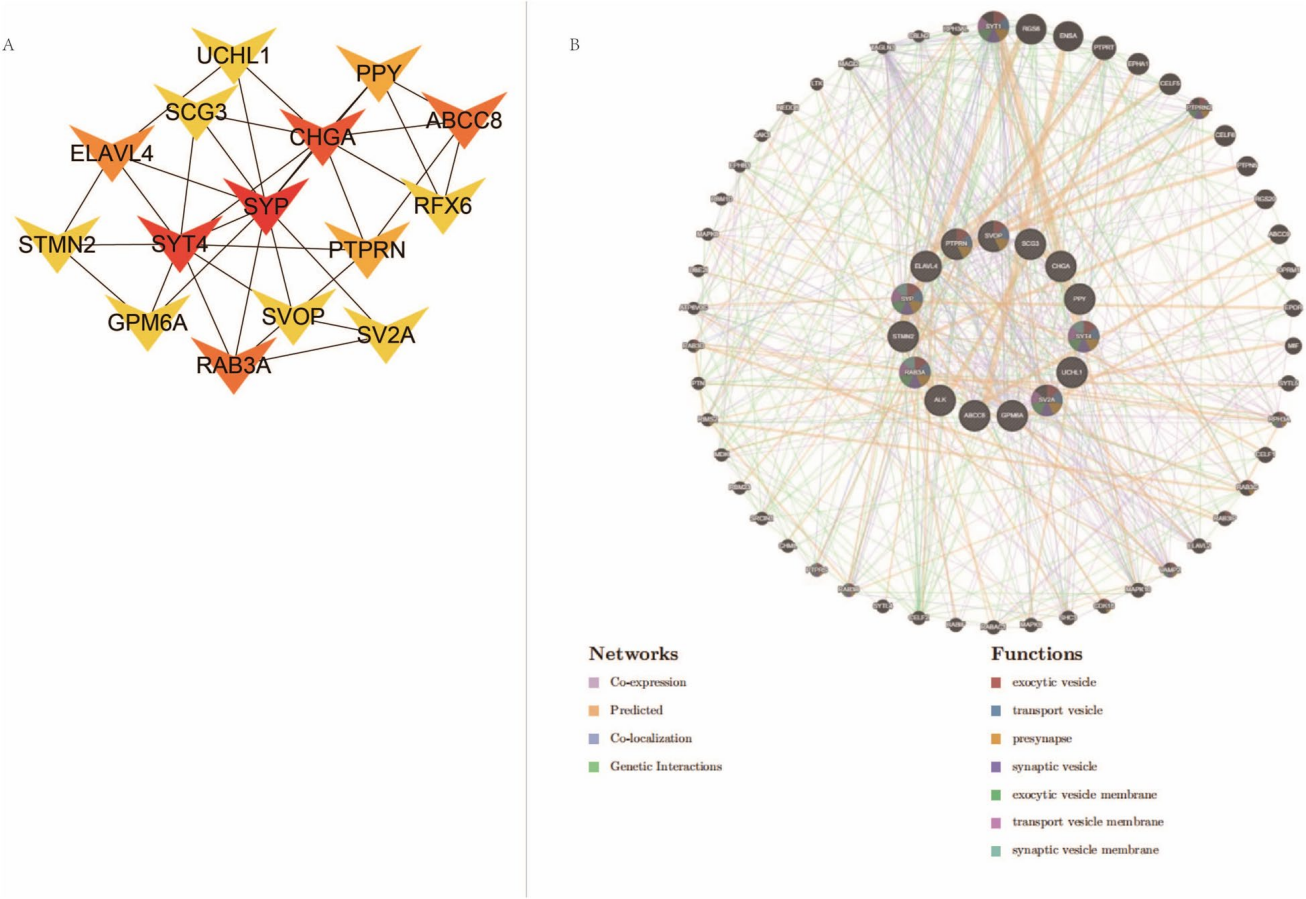
GeneMANIA prediction of hub genes. **A** Interaction network of the top 15 genes with the highest MCC scores, where deeper red indicates higher MCC scores. Arrows indicate significantly downregulated genes; **B** Functional prediction of hub genes using the GeneMANIA database, with the center part showing the 15 candidate hub genes and the periphery showing related genes obtained from GeneMANIA, with a legend below

Centrality analysis of the PPI network was conducted using the MCC method. The top 15 genes with the highest MCC scores were significantly downregulated (Fig. 5A, Supplementary Table 5), confirming their potential importance in T2DM and RC. Further analysis of these 15 candidate genes in the GeneMANIA database to explore their related genes and potential functions indicated involvement in functions like "exocytic vesicle" and "transport vesicle" (Fig. 5B, Supplementary Table 6). These functions may impact insulin metabolism and trafficking, suggesting that intercellular material or signal transduction could influence the progression of both T2DM and RC.

### Functional enrichment of hub genes

**Fig. 6:**
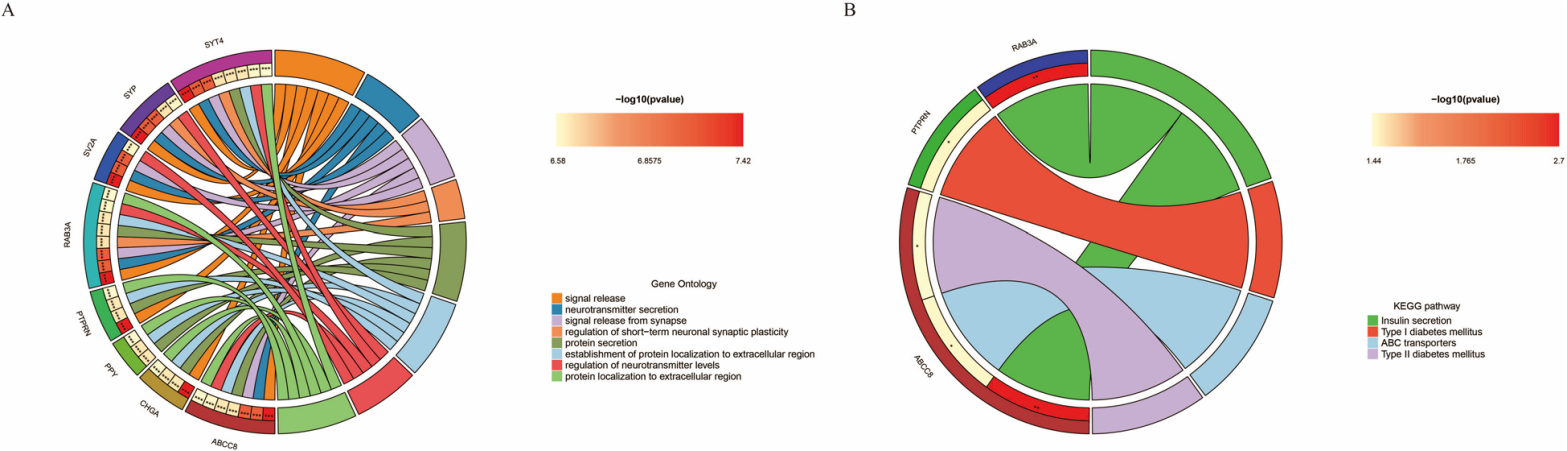
Functional enrichment analysis of hub genes. **A** Gene Ontology (GO) enrichment analysis for hub genes, with the left semicircle listing gene names and the right semicircle listing GO terms. Lines connect genes to their respective functional categories, and the histogram on the right serves as a color gradient; **B** KEGG pathway enrichment for hub genes

Further, GO and KEGG pathway enrichment was conducted on the 15 hub genes identified above. GO enrichment revealed that genes such as SYT4, SYP, SV2A, RAB3A, and PTPRN were significantly enriched in functions related to "signal release" (Fig. 6A). KEGG pathway enrichment indicated that genes like RAB3A are involved in pathways such as "Insulin secretion" (Fig. 6B). The enrichment analyses suggest that these hub genes are associated with critical cellular behaviors, including intercellular signaling and substance transfer. Notably, these genes are predominantly enriched in pathways related to insulin secretion and ATP-binding-cassette (ABC) transport, linking them closely with the development of T2DM and potentially indicating a significant overlap between the pathogeneses of T2DM and RC.

### Prediction of TFs

**Fig. 7:**
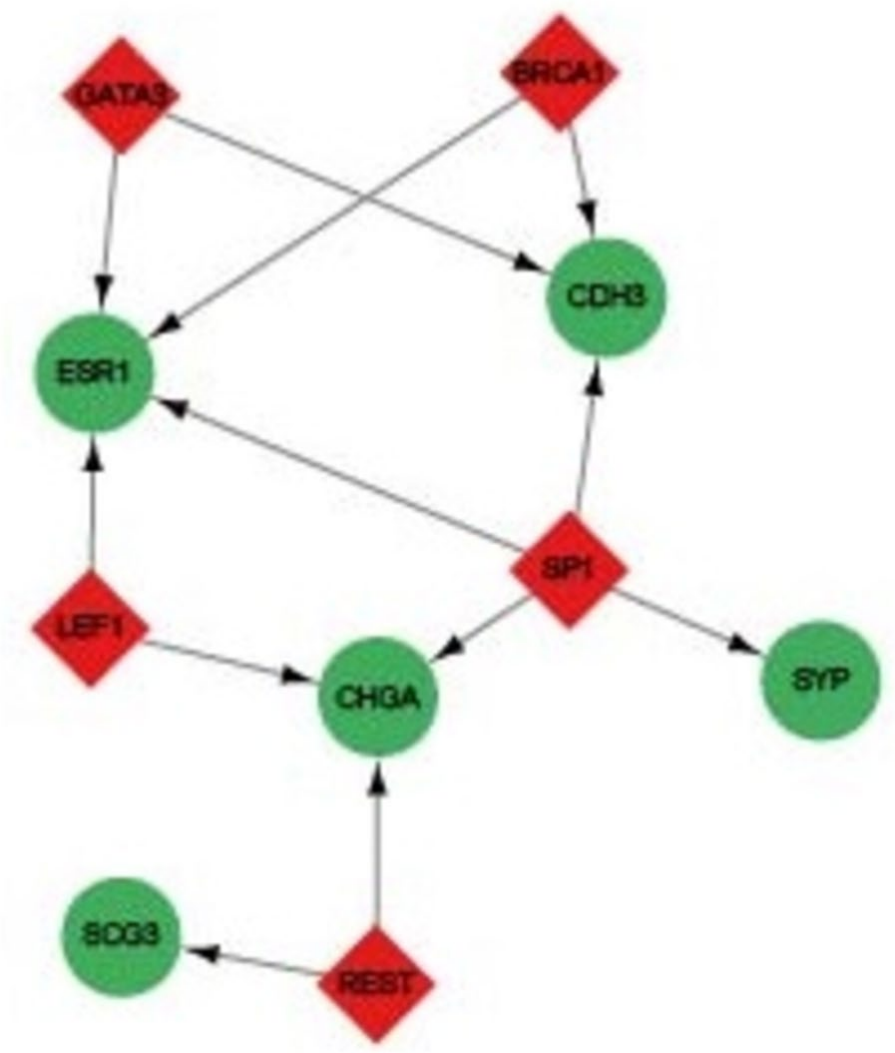
Prediction of upstream transcription factors for candidate hub genes. The figure displays predicted hub genes under transcription factor regulation as green ellipses, with red diamonds representing the predicted transcription factors. Arrows indicate regulatory relationships between the transcription factors and the candidate genes

To further understand the potential mechanisms of the identified candidate genes in T2DM and RC, predictions were made regarding the upstream TFs. The analysis indicated that genes such as ESR1, CDH3, SYP, CHGA, and SCG3 are regulated by transcription factors (Fig. 7, Supplementary Table 7), with ESR1 and CHGA being the most heavily regulated. Notably, the transcription factor SP1 is predicted to regulate CDH3, ESR1, SYP, and CHGA. Previous protein interaction network analysis identified SYP and CHGA as hub regulatory genes. The integration of protein interaction and transcription factor regulatory network analyses suggests that SP1 may play a crucial regulatory role in both T2DM and RC.

**Fig. 8:**
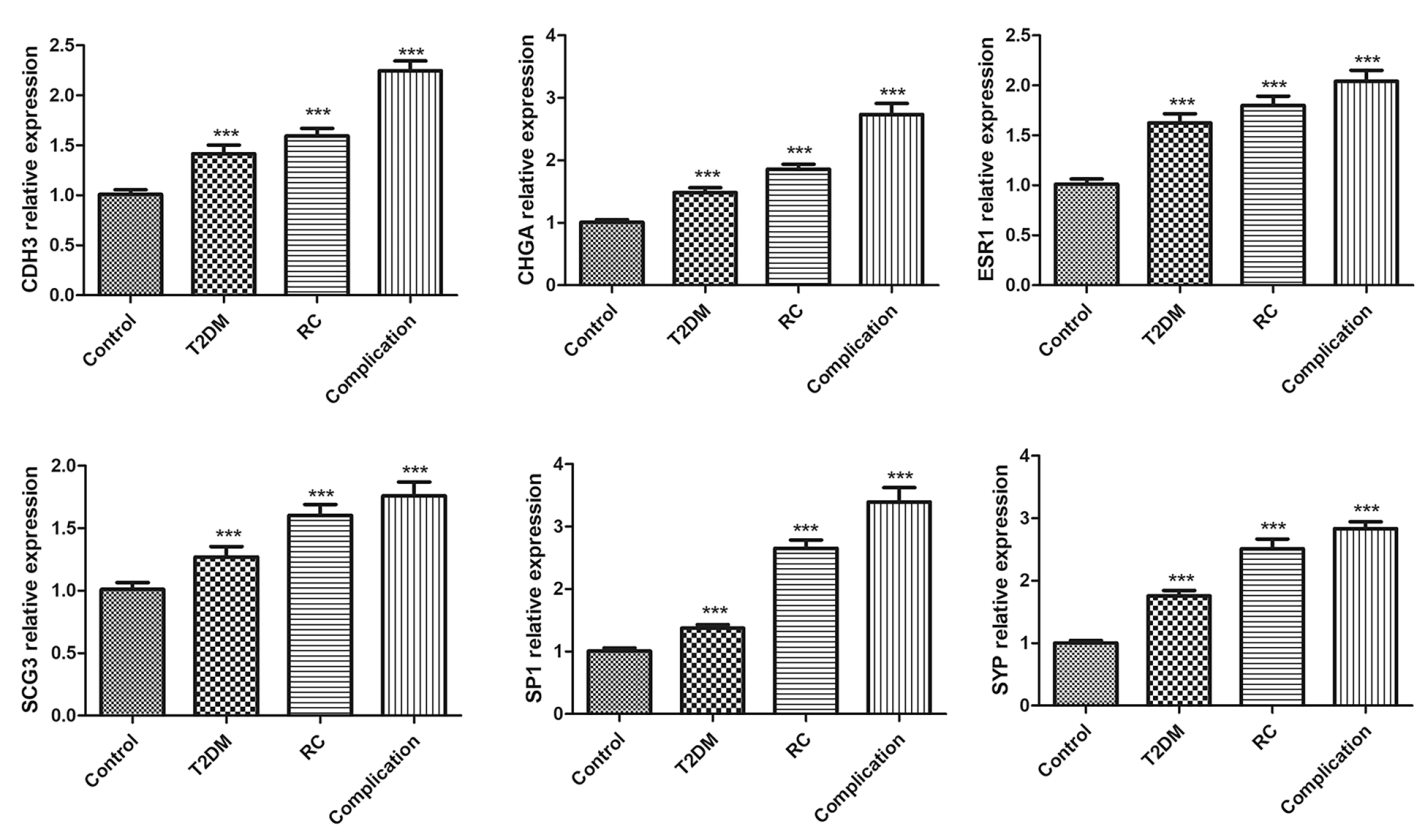
RT-PCR result of several DEG genes in T2DM and RC(n=6)

In addition, we used RT-PCR to detect the expression of several DEG genes in T2DM and RC (*n* = 6), and the results showed that CDH3, CHGA, ESR1, SCG3, SP1 and SYP genes were higher in patients with comorbidities than normal healthy people. The expressions of CDH3, CHGA, ESR1, SCG3, SP1 and SYP genes were also significantly increased in T2DM and RC (Fig. 8), which was consistent with our prediction.

### Drug prediction and molecular docking

**Fig. 9:**
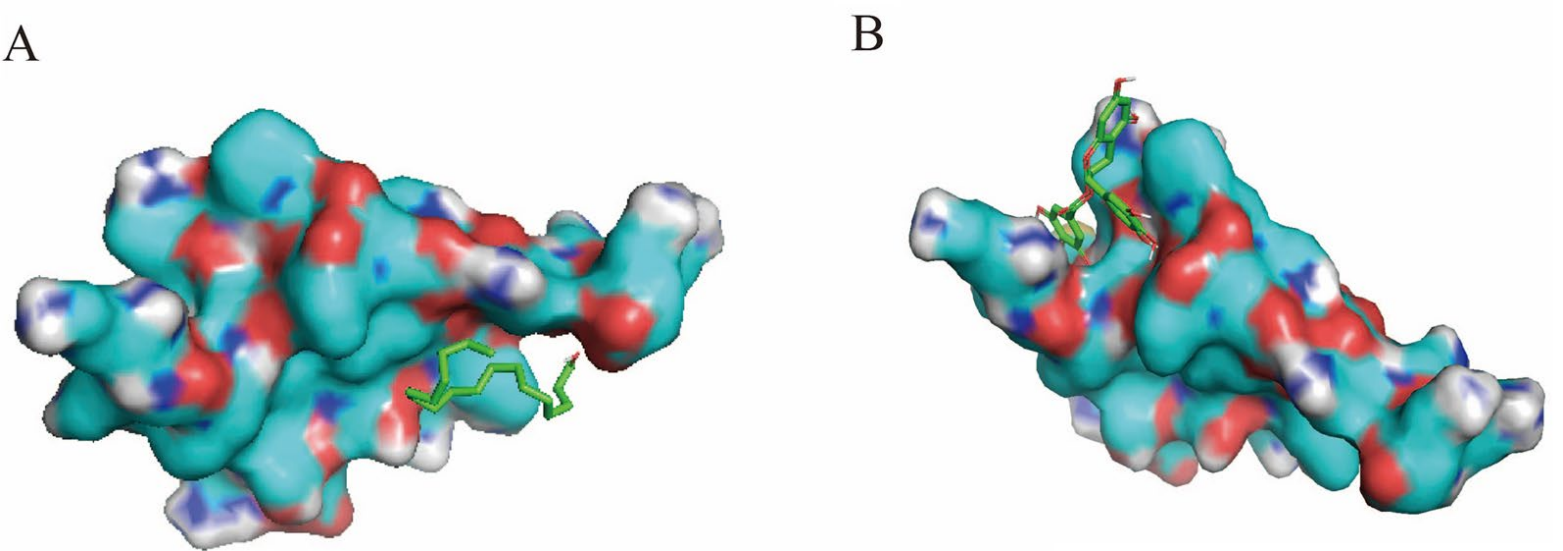
Molecular docking illustrations of SP1 with stearic acid and EGCG. **A** Molecular docking of stearic acid with SP1 (grid box parameters: center_x = 0.07, center_y = 0.149, center_z = 0.475; affinity (kcal/mol) = − 3.3). **B** Molecular docking of EGCG with SP1(grid box parameters: center_x = 0.07, center_y = 0.149, center_z = 0.475; affinity (kcal/mol) = − 7.3). The green structures represent the 2D schematic of the active drug components

To further explore the potential regulatory mechanisms of SP1 and identify potential therapeutic agents for T2DM and RC, predictive analysis was conducted on upstream regulatory drug active molecules targeting SP1. The predictions suggest that SP1 may be a target molecule for stearic acid and (-)-epigallocatechin-3-gallate (EGCG). An exploration of herbal medicines containing these compounds revealed that stearic acid is present in significant quantities in herbs like *Ginkgo biloba* and *Paeonia lactiflora*, while EGCG is primarily found in *Ginkgo biloba*, leaves of *Eriobotrya japonica*, and *Phyllanthus emblica*. Further molecular docking of these active components with SP1 suggested potential targeted binding interactions (Fig.  9 A, B). This finding implies that these components may be potential therapeutic agents for T2DM and RC. Notably, *Ginkgo biloba* contains stearic acid and EGCG, suggesting its potential as a therapeutic agent for patients with both T2DM and RC.

## Discussion

Diabetes is a hyperglycemic condition caused by multiple factors, with biochemical, molecular, and genetic characteristics. Many studies have shown that diabetes is not only a single disease but also causes other related diseases. For example, diabetic nephropathy, also known as diabetic kidney disease, is one of the main microvascular complications of diabetes. Studies have found that chicoric acid can reduce blood glucose levels and improve diabetic nephropathy by promoting the counter-regulation of PAQR3 [25]. In addition, gestational diabetes is also associated with cardiovascular diseases such as offspring hypertension, myocardial infarction, and coronary artery disease [16, 26–29]. These research results indicate that diabetes is associated with many diseases, especially a close relationship with cardiovascular diseases. Research indicates a close association between the development of T2DM and RC, with mechanisms potentially including insulin resistance and chronic inflammation [6, 30]. Moreover, physical activity has been recognized for its significant role in preventing RC [31, 32], and high levels of physical activity are closely linked to improved insulin sensitivity [33, 34]. Studies have found that exercise can effectively improve insulin resistance and aortic endothelial diastolic function in mice, thereby improving vascular endothelial function in diabetic mice [35]. In addition, high levels of physical activity also play an important role in the improvement of cardiovascular diseases. This behavior is also closely related to other physiological conditions associated with the initiation of RC, such as chronic inflammation, activity of natural killer cells, vitamin D levels, and intestinal transit time [36–39]. Additionally, statistics show that comorbidities are closely linked to poor surgical outcomes in RC patients, with diabetes being one of the most common comorbidities [40, 41]. Although numerous studies suggest a close link between diabetes and RC, the underlying regulatory mechanisms, especially in RC, still require further investigation.

As a chronic inflammatory disease, identifying diabetes-related genes is of great significance. Through bioinformatics methods, key diabetes genes can be effectively mined. For example, in the study by Huang et al., several key marker genes related to diabetes were obtained through RNA chip mining [42]. In this study, differential expression genes analysis of T2DM microarray data and RC expression data from the TCGA revealed genes differentially expressed in both diseases. We identified 17 genes that were consistently upregulated and 115 genes consistently downregulated in both T2DM and RC. This difference in the expression trend of DEGs indicates that the impact of these downregulated genes may be more critical in the comorbid diseases of T2DM and RC. Further enrichment analysis of these 128 DEGs demonstrated their primary involvement in related functional terms, such as signal release and transport vesicle. KEGG pathway enrichment analysis showed that these DEGs are mainly involved in signal pathways such as insulin secretion and TGF-β signaling pathway. It is believed that changes in insulin secretion-related genes may be more closely linked to T2DM [43–45]. The TGF signaling pathway is a well-known signaling pathway in tumors and is believed to play a key regulatory role in various tumor diseases, including RC [46, 47]. In a study about diabetes, Yadav et al. reported that changes in the TGF-β signaling pathway may be involved in regulating diabetes and obesity [48]. In addition, many researchers have found that changes in the TGF-β signaling pathway are crucial in the development of diabetes [49, 50]. It is also worth noting that some studies have reported that insulin-related signaling pathways are important in the formation of RC [51]. Pathway enrichment analysis of DEGs further suggests that there may be a close link between T2DM and RC. Further study on their commonly associated genes can provide a better understanding of the common mechanism of the two diseases. Subsequent PPI analysis of DEGs was performed on these significantly associated genes, and the hub genes in the protein interaction network diagram were searched based on the MCC calculation method. The function enrichment of the identified top 15 hub genes was performed. KEGG pathway enrichment analysis showed that the hub genes such as RAB3A were involved in related signal pathways such as insulin secretion, further suggesting that the function related to insulin secretion may not only affect the initiation and progression of T2DM but also play an important regulatory role in RC.

Subsequent predictions of transcriptional regulatory mechanisms revealed that five genes, including ESR1, CDH3, SYP, CHGA, and SCG3, are regulated by different TFs. Notably, the remaining ten genes were not predicted to be regulated by upstream TFs, suggesting the presence of multiple molecular regulatory mechanisms in T2DM and RC. This finding also indicates that the potential association mechanisms between T2DM and RC may be highly complex and require further study. Among these TF-regulated genes, ESR1 and CHGA are controlled by the highest number of TFs, implying that these two genes may be more critical in the regulation related to T2DM and RC. Several studies have suggested that the ESR1 and its associated signaling pathways play important regulatory roles in the development of RC [52, 53]. In diabetes and obesity-related diseases, ESR1 is also considered to have significant regulatory effects [54, 55]. In diabetes, CHGA is regarded as one of the biomarkers [56] and is considered a key regulatory gene related to the development and regulation of diabetes in many studies [57–59]. CHGA is also deemed a crucial regulatory factor in tumors [60]. For instance, in prostate cancer, CHGA is considered an important biomarker [61, 62]. Kim et al. also found that changes in CHGA expression are significantly associated with the prognosis of patients with rectal cancer [63]. These results indicate that in the development of T2DM and RC, especially in their comorbidity, ESR1 and CHGA may play pivotal regulatory roles.

Among the potential regulatory transcription factors, SP1 is noteworthy. It is predicted not only to regulate ESR1 and CHGA but may also transcriptionally regulate CDH3 and SYP. SP1, as a transcription factor, has been found to have potential as a tumor chemotherapy target gene [64]. Moreover, in various tumors such as prostate cancer, non-small cell lung cancer, and cervical cancer, SP1 is considered to play a significant regulatory role [65–69]. In diabetes, alterations in SP1-related pathways are considered one of the important reasons for disease development [70–72]. A further prediction of potential upstream targeting drugs for SP1 revealed that stearic acid and EGCG are two active compounds that may target SP1. Notably, the TCMSP database shows that the herbal medicine *Ginkgo biloba* contains stearic acid and EGCG [73]. In several studies, *Ginkgo biloba* is considered an effective therapeutic agent for T2DM [74, 75]. Similarly, in non-small cell lung cancer and colorectal cancer, *Ginkgo biloba* has also been found to have therapeutic potential [76, 77]. In this study, through molecular docking predictions between stearic acid and EGCG and the SP1 protein, we found that these compounds likely bind to SP1 and consequently affect its function. This finding suggests that *Ginkgo biloba* may have potential as a therapeutic agent for patients with comorbid T2DM and RC. These results provide a theoretical basis for further understanding the potential regulatory mechanisms of *Ginkgo biloba* in T2DM and RC and offer new insights for the discovery of therapeutic drugs for these diseases.

Although this study provides new insights for further understanding the shared regulatory mechanisms of T2D and RC and for drug selection, it still has certain limitations. First, the main data in this study are all from public databases. In addition, the therapeutic properties of the predicted drugs still require further clinical trials to confirm their efficacy. Nevertheless, our results can serve as a basis for future research on the regulation and treatment of T2D and RC.

## Conclusion

In summary, genes such as SYP may play significant roles in the progression of T2DM and RC. Transcription factor analysis suggested that SP1 could regulate multiple hub genes. Molecular docking indicated that SP1 is a potential target for active components like stearic acid and EGCG in *Ginkgo biloba*, highlighting its potential as a therapeutic agent for T2DM and RC.

## Supplementary Information

Supplementary material 1. Table 1 DEGs of GSE76894

Supplementary material 2. Table 2 DEGs of TCGA

Supplementary material 3. Table 3 GO enrichment analysis results

Supplementary material 4. Table 4 KEGG enrichment analysis results

Supplementary material 5. Table 5 The top 15 MCC score of PPI

Supplementary material 6. Table 6 GeneMANIA database prediction results

Supplementary material 7

## Data Availability

The datasets used and analyzed during the current study are available from the corresponding author upon reasonable request.

## Abbreviations

T2DM: Type 2 diabetes mellitus
CRC: Colorectal cancer
RC: Rectal cancer
GEO: Gene expression omnibus
DEGs: Differentially expressed genes
EGCG: (-)-Epigallocatechin-3-gallate
PPI: Protein–protein interaction
TFs: Transcription factors
PECAM-1: Platelet-endothelial cell adhesion molecule 1
FDR: False discovery rate
GO: Gene ontology
KEGG: Kyoto encyclopedia of genes and genomes
CC: Cellular components
BP: Biological processes
MF: Molecular functions
MCC: Maximal clique centrality
ABC: ATP-binding-cassette
